# Eating disorder symptoms and foraging for food related items

**DOI:** 10.1186/s40337-021-00373-0

**Published:** 2021-02-10

**Authors:** Árni Kristjánsson, Auður Helgadóttir, Tómas Kristjánsson

**Affiliations:** 1 Faculty of Psychology, University of Iceland, Nýi Garður, 101 Reykjavík, Iceland; 2 School of Psychology, National Research University Higher School of Economics, Moscow, 101000 Russia

**Keywords:** Eating disorders, Anorexia nervosa, Bulimia nervosa, Visual attention, Attention Bias, Foraging

## Abstract

**Background:**

Foraging tasks have recently been increasingly used to investigate visual attention. Visual attention can be biased when certain stimuli capture our attention, especially threatening or anxiety-provoking stimuli, but such effects have not been addressed in foraging studies.

**Methods:**

We measured potential attentional bias associated with eating disorder symptoms to food related stimuli with our previously developed iPad foraging task. Forty-four participants performed a foraging task where they were instructed to tap predesignated food related targets (healthy and unhealthy) and other non-food objects and completed four self-report questionnaires measuring symptoms of eating disorders. Participants were split into two groups based on their questionnaire scores, a symptom group and no symptom group.

**Results:**

The foraging results suggest that there are differences between the groups on switch costs and target selection times (intertarget times) but they were only statistically significant when extreme-group analyses (EGA) were used. There were also notable food versus non-food category effects in the foraging patterns.

**Conclusions:**

The results suggest that foraging tasks of this sort can be used to assess attentional biases and we also speculate that they may eventually be used to treat them through attention bias modification. Additionally, the category effects that we see between food items and other items are highly interesting and encouraging. At the same time, task sensitivity will need to be improved. Finally, future tests of clinical samples could provide a clearer picture of the effects of eating disorder symptoms on foraging for food.

## Plain English summary

A common symptom of many anxiety disorders is that attention is preferentially drawn towards anxiety provoking stimuli. We measured such attention biases in eating disorders by investigating foraging for healthy and unhealthy foods and objects unrelated to food, while we also measured participants eating disorder symptoms on 4 clinical diagnostic scales. The results showed that foraging performance varied as a function of the strength of eating disorder symptoms. This pattern was, however, only statistically significant when extreme group analyses were used. These results may suggest that symptoms of eating disorders lead to attentional biases and we speculate that foraging tasks are a promising tool for assessing and treating attentional biases in eating disorders, through attention bias modification.

## Introduction

*See my third rib appear; A week later all my flesh disappear; Stretching taut, cling-film on bone; I’m getting better.*

*I want to walk in the snow; And not leave a footprint. *from 4st 7 lb., Manic Street Preachers.

At every waking moment hundreds, if not thousands of different things compete for our attention. Various mechanisms enable us to focus attention on the things that matter most to us such as recognizing our child among other children in the park or finding our favorite candy bar at the convenience store. Even though the items we look for are embedded within many similar items, we still manage to find our targets, most often rather easily. Such orienting within the visual environment has typically been investigated with visual search tasks (see [30, 37, 38, 41, 66]; for critical reviews) where the goal is to search for a particular target.

An example of visual search is when animals locate food in the wild. But there are often many food targets to choose from and animals often select many targets in sequence, a task typically referred to as *foraging*. During such foraging tasks, when targets are hard to detect, animals may focus on a single food source and ignore other available sources, selecting the same target type in non-random sequences that are longer than can be expected by chance, also called *runs.* But if prey can be easily detected the animal may switch effortlessly between different prey types [9, 15, 16, 61]. Investigations of foraging may better capture attending in dynamic visual environments than single target visual search tasks since our goals do not usually involve a single target and there may be many items to look out for in any given environment.

Foraging has been investigated extensively in animals but is a fairly recent topic in studies of attentional orienting in humans. An early example is Bond [8] where participants were asked to sort different colored beads as fast as possible. Participants who sorted the beads in non-random sequences were fastest and most accurate at performing the task. More recently, Kristjánsson, Jóhannesson and Thornton [30] introduced an iPad foraging task to measure human foraging. Their aim was to gain a better understanding of how participants orient visual attention when they search for multiple targets from different categories [30]. Their task had two different conditions - feature foraging where only one feature distinguishes targets from distractors (e.g. color), and conjunction foraging where targets differ from distractors on two features (e.g. color and shape). They found that during easy foraging, participants had no trouble switching between target categories but when the task was harder (during conjunction foraging) participants tended to exhaust one category in long runs before switching to the next one. Kristjánsson et al. [30] concluded that this run-like behavior was due to differences in attentional load (see also [56]). Other aspects of performance that differed between difficulty conditions involved the costs of switching between target categories and intertarget times (T [38]). Other aspects of human foraging that have been investigated include how humans organize their foraging (e.g. [59, 60, 68]), how it relates to working memory (e.g. [31]) and how the availability and quality of visual information (e.g. [65]) and the value of the items we forage (e.g. [66]) affect attentional orienting.

### Attentional bias

Our attention is often biased to targets that match our intentions [17, 35]. These could be items of particularly high value [36, 49];), items that are highly important for current behavior [67] or have been primed through repeated selection ([11, 48]; see [29] for review).

Our attentional preferences seem in other words to be influenced by motivation. But our motivations can also be biased by fear or anxiety (see [6] for review) that may even reflect pathological conditions such as social anxiety disorder or depression [4]. Results on how we attend to threatening stimuli suggest that clinically anxious individuals selectively attend to threatening information (for a review see [63]). According to Beck [5], anxious individuals have cognitive “schemas” associated with threat that direct attention to threatening stimuli. Clark [12] proposed that different anxiety disorders are associated with different types of attentional bias. Selective attention towards or away from particular threatening stimuli will be determined by the specific phobias or anxiety disorders in each case.

How can such attentional biases be measured? One measure involves the classic Stroop [54] task where observers must report the color of a written word while ignoring its semantic meaning that can be related to their anxiety [40]. Other examples involve the dot-probe [39], visual search [69] and spatial cueing tasks [32]. These tasks are widely used and some studies have suggested that they are useful for measuring abnormal attention bias (see review in [4]). Recently, however, we reported that the tasks most often used for measuring and modifying attentional bias are not particularly sensitive to such biases [49, 50]. More encouragingly we also reported that a novel task based on the attentional blink task [33, 44] involving different facial expressions, discriminated well between participants with low versus high anxiety. Importantly, Sigurjónsdóttir et al. [51] replicated this in a clinical sample of patients with social anxiety disorder.

### Eating disorders and attentional bias

Eating disorders are highly comorbid with anxiety [27, 55] and are characterized by a persistent disturbance in eating behavior, that affects caloric intake, physical health and/or psychosocial functioning. Anorexia Nervosa (AN) and Bulimia Nervosa (BN) are among the most common eating disorders. Anorexia Nervosa is characterized by starvation where caloric intake is severely restricted, eventually leading to very low bodyweight, a low body mass index (BMI), and a distortion in self-perception of the size and shape of the body and an over-emphasis on body shape and size in self-evaluations [1]. Bulimia Nervosa is characterized by frequent binges where a large amount of food is eaten over a short period of time, accompanied by compensatory behavior to prevent weight gain. These may include self-induced vomiting, laxative use, enemas, fasting or over-training. As in AN there is an over emphasis on body shape and size in self-evaluations and distorted self-perception [1]. AN and BN are serious disorders with high mortality rates, from medical complications, suicide or substance abuse [2]. While the prevalence of these disorders in their most serious forms is not high, many core characteristics are widely found in nonclinical populations. Being unsatisfied with body shape and size, ashamed over amounts of food eaten or being on a strict diet is something that many healthy individuals can relate to. Even though most participants (in general populations) score well within the non-clinical range, importantly there is considerable variance in participant scores [7, 19, 23].

Cognitive biases in the etiology and maintenance of eating disorders reveal that maladaptive cognitive schemas can cause biases in the allocation of attention, memory and interpretation of new information [18, 64]. Modified Stroop tests have been widely used to measure attention bias in eating disorders (see e.g. [18, 20] for review). A meta-analysis [18] suggests that BN patients show consistent attentional bias on the Stroop task across a range of stimuli but the results were limited to body and weight stimuli for AN participants, consistent with core diagnostic features of the disorders.

Other methods of measuring attentional bias have been used. Shafran, Lee, Cooper, Palmer & Fairburn [47] found that patients with eating disorders were quicker to respond when a probe appeared in the same location as a food stimulus that experts in the field rated as inducing a negative emotional response and slower when a probe appeared in the same location as a food stimulus rated as inducing a positive response. These biases were greater than for anxious individuals and normal controls. Similar biases were found for weight and shape related words by Rieger et al. [45].

Attention biases have also been measured with visual search tasks. Schmidt, Lüthold, Kittel, Tetzlaff & Hilbert [46] tested adolescents with binge-eating disorder on a visual search task with food and non-food items finding that the binge-eating group showed a greater detection bias for food targets than controls, as well as differing gaze characteristics. Smeets, Roefs, Furth & Jansen [52] tested visual search for food and body related words finding speeded detection of body-related information, and increased distraction by food related words for patients with eating disorders. Brand, Masterson, Emond, Lansigan and Gilbert-Diamond [10] tested attention biases to food in children aged 3–6 years using visual search with eye-tracking measures finding that food related items seemed to capture attention more strongly than other items, especially for children with a high body/mass index (BMI). Additionally, other reports of attention biases related to eating disorders have been found with visual search related tasks (e.g. [3, 53, 57]), but no studies have yet tested such attention biases with foraging tasks. Foraging tasks yield a number of different dependent variables (for example, run number, intertarget selection times, error rates, selection strategies and more) that may increase their value above other attention tasks such as visual search (see e.g. T [37, 38].; and an overview in [34]).

### The current study

Our aim was to measure potential attentional biases connected with eating disorder symptoms with our newly developed iPad foraging task (Á [30, 37, 38].) in a non-clinical sample. The foraging task has proved to be highly sensitive to various aspects of attentional dynamics, as discussed above. Participants answered four self-report questionnaires that measure symptoms of eating disorders and foraged through either healthy or unhealthy food items among non-food objects. We measured the relationship between visual attention and symptoms of eating disorders in a non-clinical sample, but the results could potentially be used to develop treatments of attentional bias in eating disorders although we emphasize that any such tasks will require considerable further development. Foraging tasks have to our knowledge not been previously used to measure visual attention in the context of anxiety.

We assessed whether foraging of participants with eating disorder symptoms differs from those without such symptoms. For example, we expected that those with eating disorder symptoms might allocate their attention toward food stimuli more often or more strongly than those with no symptoms, therefore having lower intertarget selection times, or those with symptoms might refrain from focusing on the food stimuli, with higher intertarget times. We also expected observers with eating disorder symptoms to have more trouble disengaging attention from food related items which could cause two patterns of results: firstly higher interselection times and secondly, that the run numbers for food related items would be lower. Another related aim was to assess whether scores on self-report questionnaires for signs of eating disorders could predict foraging performance.

## Method

### Participants

Forty-four participants were recruited for the foraging task (32 women, 12 men; mean age = 24.8 (SD = 3.5), ranging from 21 to 42 years). Thirty-one were recruited from a course in cognitive psychology where participating in research was a course requirement, the rest were volunteers. All had normal or corrected-to-normal vision and gave written, informed consent. Because of the nature of the data collection, we did not have any means of controlling how hungry our participants were at the time of testing, or what sort of food they ate before testing. The experiment was reviewed and approved by the National Bioethics Committee of Iceland and the Data Protection Authority of Iceland (# 18–032).

### Equipment

The foraging task was performed on an iPad Air with screen dimensions of 20 × 15 centimeters and an effective resolution of 2048 × 1536 pixels. Viewing distance was approximately 40 cm. A custom application written in Swift using Xcode presented the stimuli and collected the data.

### Self-report questionnaires

*The Body Shape Questionnaire (BSQ*, Cooper, Taylor, [14, 26]) is a self-report questionnaire measuring respondents satisfaction with their own body, concerns about body shape and the experience of “feeling fat” over the last 4 weeks. The questionnaire consists of 34 items that respondents answer on a six point Likert scale, from never (1) to always (6). Total scores range from 34 to 204. In Cooper et al. [13] a group of patients with eating disorders had a mean score of 136.9 (chosen as the cut-off score and SD = 22.5), probable cases had a mean score of 129.3 (SD = 17) and definite non-cases had a mean score of 71.9 (SD = 23.6 [13];). Cooper et al. argue that the BSQ measures a psychological dimension found both in patients and community samples and that the questionnaire should therefore typically be used to measure the extent of psychopathology rather than as a binary diagnostic measure. The reliability of the BSQ was excellent in the Icelandic population, both for in-patient (⍺ = 0.95) and student samples (⍺ = 0.97 [70];). The Icelandic version had good internal consistency (α = 0.81) in this study.

*The Bulimia Test – Revised (BULIT-R)* is a self-report questionnaire that measures core symptoms of Bulimia Nervosa; binge eating, purging and worries about body shape and size. The items consist of 36 different statements (e.g. „I am satisfied with the shape and size of my body“) and questions (e.g. “Compared with people your age, how preoccupied are you about your weight and shape?”) that respondents answer on different scales ranging from 1 to 5. The total score is between 28 and 140 with a 104 cut-off score ([58]; Icelandic translation by [26]). Reliability reported in Gunnarsdóttir [25] was excellent (⍺ > 0.95 in a student sample) and the validity is good [26]. In our study the internal consistency (α = 0.79) was acceptable.

The *Eating Disorder Examination Questionnaire (EDE-Q)* is a 28 item self-report version of the Eating Disorder Examination (EDE) interview, developed in 1987 [14]. The items evaluate four core characteristics of eating disorders: restraint, eating concern, shape concern and weight concern. A total score is computed from the questionnaire and a separate score for each subscale. In Ólafsdóttir [42] the mean total score was 3.13 in an in-patient sample and 1.64 in a student sample the cut-off score is indicated as ~ 4, although this entails various caveats [14, 42]. The sixth version of the EDE-Q was used in the Icelandic translation [42]. The reliability was excellent (⍺ = 0.90 in a clinical sample, ⍺ = 0.91 in student sample) with good concurrent and discriminant validity [42], and in our study the internal consistency (α = 0.85) was good.

*Binge Eating Scale (BES):* The Binge Eating Scale is a 16 item (each item ranging from 1 to 3, so the scores can range from 0 to 47) self-report questionnaire measuring severity of binge eating in obese adults. The questionnaire was originally developed by [24]; Icelandic version, [62], typical cut-off score = 27). The Icelandic version has good reliability (⍺ =0.87 in a clinical sample, ⍺ =0.88 in a comparison sample) and good concurrent and discriminant validity [62] and here the internal consistency (α = 0.82) was good.

### Foraging stimuli

**Fig. 1:**
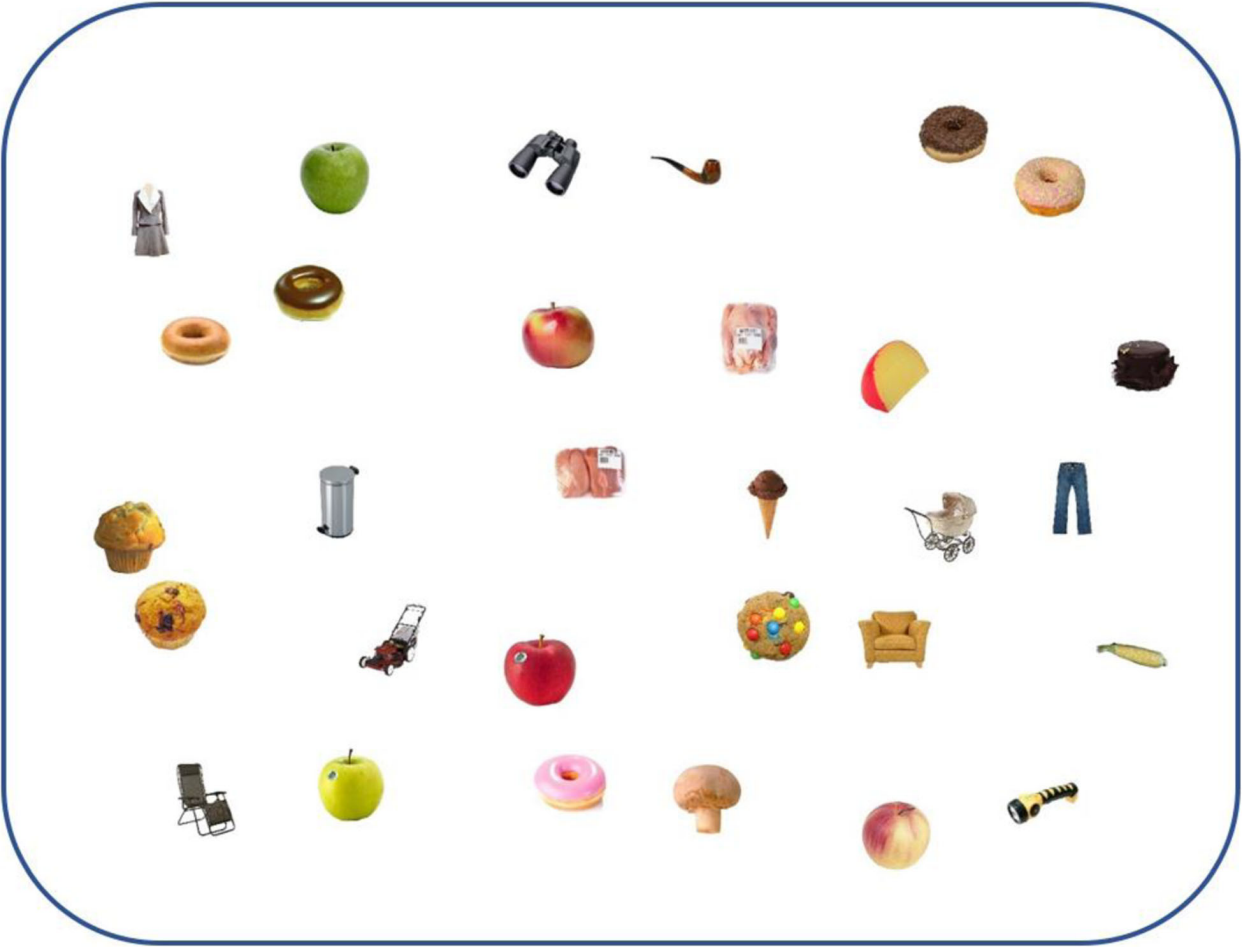
An example of a trial in the iPad foraging task with 10 healthy, 10 unhealthy items and 10 objects

We used 30 images from three stimulus categories, ten each of healthy and unhealthy food items and non-food items (objects). All came from the stimulus set provided by Konkle, Brady, Alvarez and Oliva [28]. Over 200 stimuli were pre-selected and pre-tested by 11 participants (who did not participate in the main study) to identify the ten healthy and ten unhealthy food items that respondents were quickest to recognize as food and give a health rating between 1 (very healthy) and 9 (very unhealthy) in addition to the ten objects that respondents were quickest to recognize as objects. The items were randomly distributed on the iPad screen across a non-visible 6 × 5 grid, offset from the screen edge by 114 × 110 pixels. Stimulus position within the grid was jittered by adding a horizontal and vertical offset to create less uniform appearance (see Fig. 1). Layout and location were generated independently on every trial.

### Design

We measured 3 dependent variables from the foraging task, (i) *the inter target time (ITT)*: the time between taps on targets in milliseconds; (ii) *run number*: how often targets within the same stimulus category are sequentially selected, for example if a healthy target is selected three times and an unhealthy target then selected twice, this is defined as two runs. The minimum run number is therefore 2 (if all stimuli in one target category are tapped before moving on to the next category) and 20 at maximum (if observers switch categories for each target selection); (iii) switch-costs, the difference between intertarget times when participants select from the same category or switch to the other one.

To test whether participants differed on the dependent variables based on their score on the self-report questionnaires, the sample was split into two groups. Participants with a score as high as, or higher than the designated cut-off score on any of the self-report scales were placed in the “symptom “group, others in the “no-symptom “group. We reasoned that these participants were more likely than the others to show some symptoms of eating disorders. As an exploratory analysis, we also conducted extreme group analyses (EGA), in an attempt to assess whether participants at the extremes on the psychometric measures showed the largest effects in either direction. While EGA is an established method [21, 43] we do emphasize that any conclusions from such analyses should only be taken in the intended spirit – as hints about what the data may reveal, and as food for thought for further experimentation, but not as results of large significance regarding attentional symptoms in eating disorders.

### Procedure

The experiment was run in a quiet room with normal illumination. Participants either started by answering the self-report questionnaires or completing the foraging task in counter-balanced order. For the foraging task participants had to tap all of the items in two of the categories (20 stimuli) and ignore one category (10 stimuli). Participants finished sixteen foraging trials (with 20 targets in each) in the 3 different conditions in counter-balanced order (48 trials per participant). The 3 conditions were: tap healthy and unhealthy food items but ignore objects (H/U-O), tap unhealthy foods and objects but ignore healthy food (U/O-H), tap healthy food and objects but ignore unhealthy food (H/O-U). Participants were asked to tap all targets as quickly as possible while avoiding mistakes, using the index finger of their dominant hand. When tapped, the items immediately disappeared. Once all the targets were selected the trial ended and participants received text feedback on how quickly they finished the trial and whether they had responded correctly. If a distractor item was tapped an error message was given and participants repeated that trial with a new layout.

### Data analysis

Repeated measures ANOVAs (alpha level = .05) compared mean performance across conditions and groups (Greenhouse-Geisser corrections for *p*-values used when sphericity was violated). A stepwise multiple regression analysis assessed whether scores on the self-report questionnaires predicted performance on the iPad foraging task (alpha = .05).

## Results

Table 1 shows error rates, mean intertarget times (ITT), run numbers and switch-costs in each condition for the total sample. A repeated measures ANOVA revealed a significant effect of condition on ITT’s (*F*(2,86) = 301.96, *p* < .001, η_p_^2^ = .875), run number (*F*(2,86) = 40.46, *p* < .001, η_p_^2^ = .485) and switch-costs (*F*(2,86) = 36.75, *p* < .001, η_p_^2^ = .461). There was a significant interaction between condition and switch-costs (*F*(1,43) = 36.75, *p* < .001, η_p_^2^ = .461). Error rates were lowest in the H/U-O condition, roughly half the errors made in the H/O-U and U/O-H conditions.

**Table 1:** Total errors, inter target times, number of runs and switch costs in each condition

	Errors	Inter-target time (*SD*)	Number of runs (*SD*)	Switch-cost (*SD*)
H/U-O	79 (20%)	412.4 ms (78.3)	10.7 (*.47*)	9.15 ms (*36.9*)
H/O-U	144 (36.5%)	601.1 ms (94.6)	9.3 (*.99*)	96.4 ms (*76.7*)
U/O-H	171 (43.5%)	612.2 ms (91.1)	9.2 (*1.2*)	114.9 ms (*86.7*)

### Eating disorder symptoms and foraging

Table 2 shows the cut-off scores, mean scores and standard deviation for each self-report questionnaire. Sixty-seven values were over the designated cut-off yielding 20 participants for the symptom-group. To be included in the symptoms groups, observers had to score above the cut-off score on one or more of the measures. The symptom group included 20 participants (16 females and 4 males, mean age 25.3), while the non-symptom group included 24 participants (16 females and 8 males, mean age 24.1).

**Table 2:** Results from self-report questionnaires

	N	Cut-off score	Minimum	Maximum	Mean	Standard deviation	N over cut-off
BSQ	42	110	38	158	86.2	29.7	7
BES	41	17	0	26	10.2	6.5	7
BULIT-R	44	104	32	104	49.4	17.2	1
EDE-Q Total	44	2	.15	4.2	1.5	1.1	13
EDE-Q Restraint	44	2	0	4	1.3	1.1	14
EDE-Q Eating Concerns	44	2	0	3.6	.6	.9	4
EDE-Q Shape concerns	44	3	.1	5.3	2.15	1.4	11
EDE-Q Weight concerns	44	3	0	5.2	1.9	1.4	10

#### Inter-target times

**Fig. 2:**
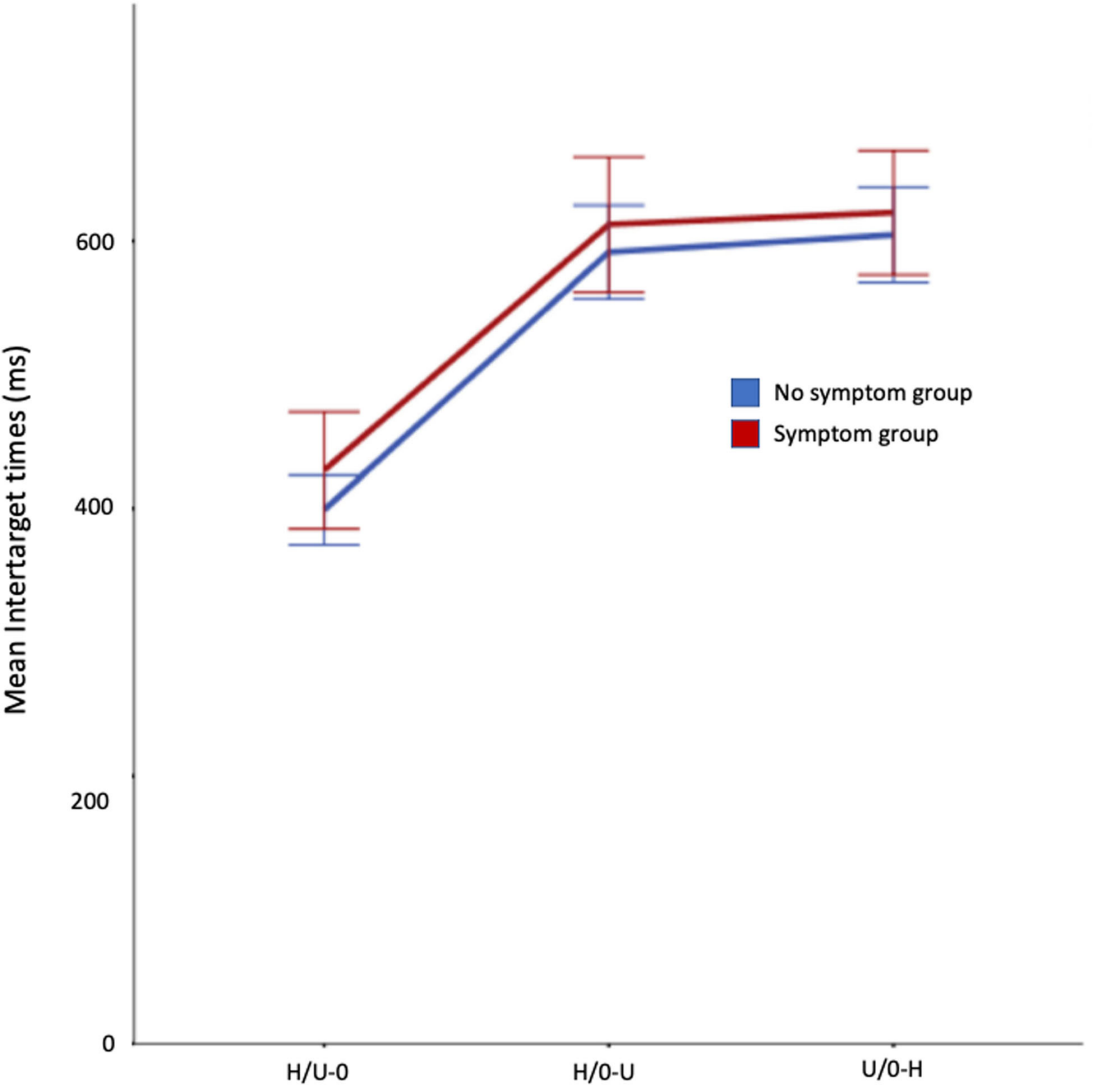
Mean ITTs by condition and symptom groups

The mean ITT’s in each condition in both groups are shown in Fig. 2. Although the symptom group had slightly higher mean ITT’s than the no-symptom group in all conditions, this was not statistically significant. Both groups had the shortest mean ITT’s in the H/U-O condition (where observers selected food items but were instructed to ignore objects) and longest in the O/U-H condition. A 3 × 2 (condition x symptom group) repeated measures ANOVA showed a significant main effect of condition (*F*(2, 84) = 292.72, *p* < .001, η_p_^2^ = .875) but not of symptom group (*F*(1, 42) = .827, *p* = .37, η_p_^2^ = .02) nor any interaction between condition and symptom group (*F*(2, 84) = .248, *p* = .78, η_p_^2^ = .006).

#### Run numbers

**Fig. 3:**
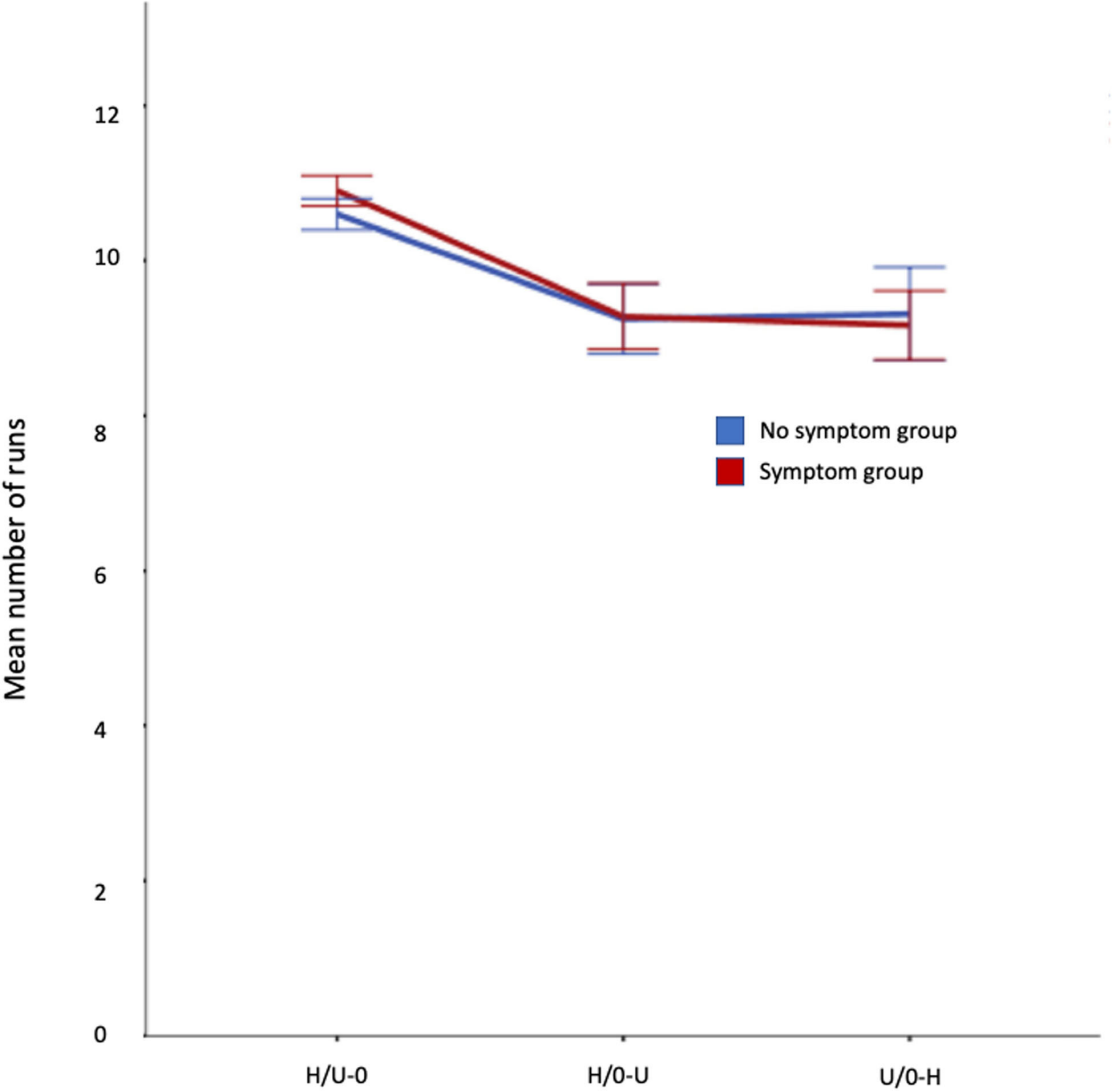
Mean run numbers by condition and symptom group

Figure 3 shows that run numbers are high in both groups in all conditions suggesting that participants switched between target categories without much difficulty. Mean run number is, perhaps, unsurprisingly highest in the H/U-O condition (where observers selected food items but were instructed to ignore objects) for both groups, since intertarget times were also shortest in this condition. A 3 × 2 (condition x symptom group) repeated measures ANOVA revealed a significant main effect of condition (*F*(2,84) = 40.727, *p* < .001, η_p_^2^ = .492) but not of symptom group (*F*(1,42) = .118, *p* = .733, η_p_^2^ = .003). The interaction between condition and symptom group was not significant (*F*(2,84) = .696, *p* = .501, η_p_^2^ = .016).

#### Switch-costs

**Fig. 4:**
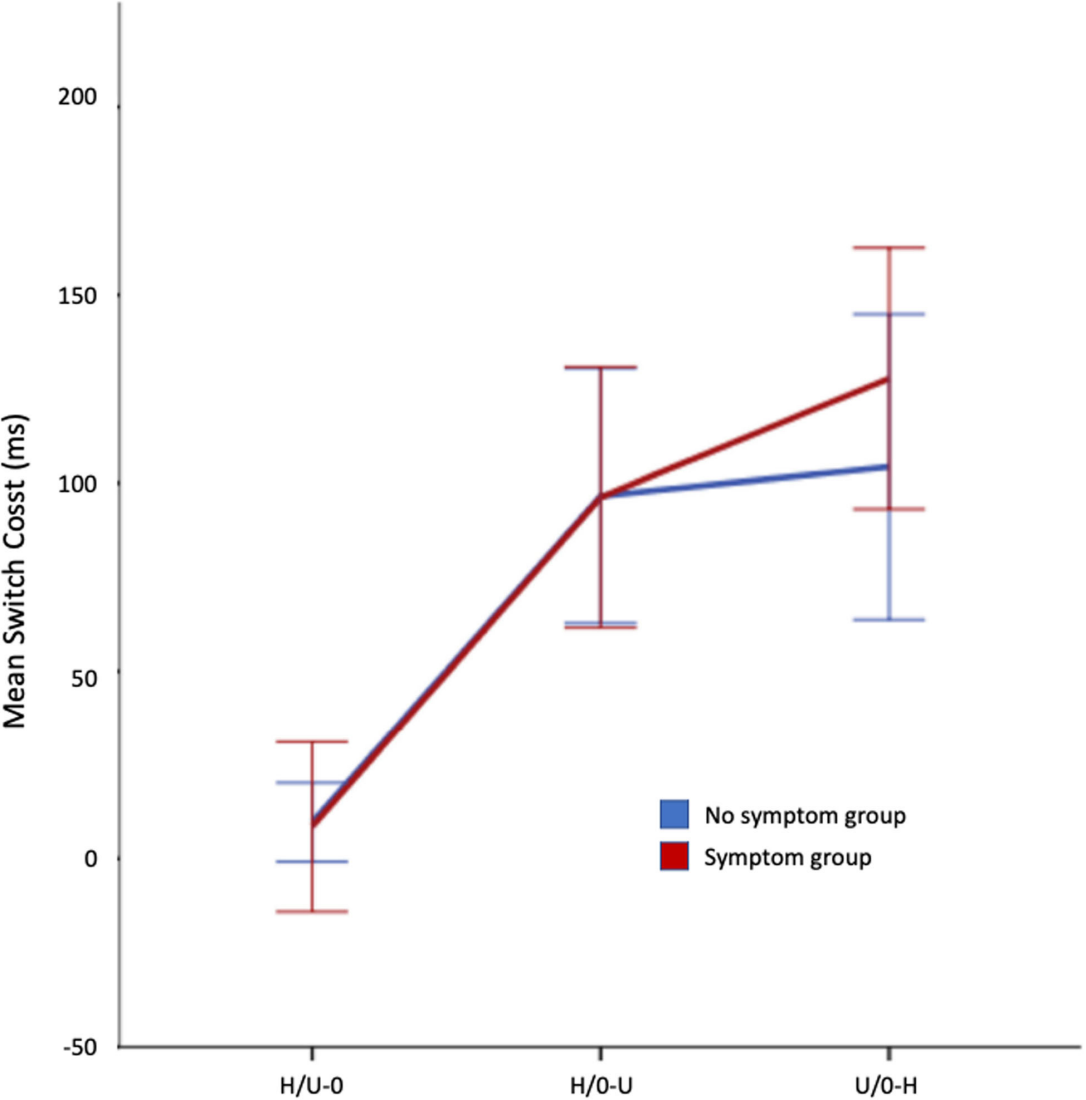
Mean switch-cost by conditions and symptom groups

Mean switch-costs for both groups in all conditions are shown in Fig. 4. The switch-costs were markedly lower in the H/U-O condition than in the other two conditions. Once again this probably reflects that the targets in this condition were food items while observers were to ignore the objects. Both the symptom group and no-symptom group showed similar switch-costs in the H/O-U and the H/U-O conditions. A 3 × 2 (condition x symptom group) repeated measures ANOVA revealed a significant main effect of condition (*F*(2,84) = 36.6, *p* < .001, η_p_^2^ = .466) but not of symptom group (*F*(1,42) = .118, *p* = .242, η_p_^2^ = .006). Numerically, the difference between the two groups was largest in the O/U-H condition but the interaction between condition and group was not significant (*F*(2,84) = .556, *p* = .576, η_p_^2^ = .013).

**Fig. 5:**
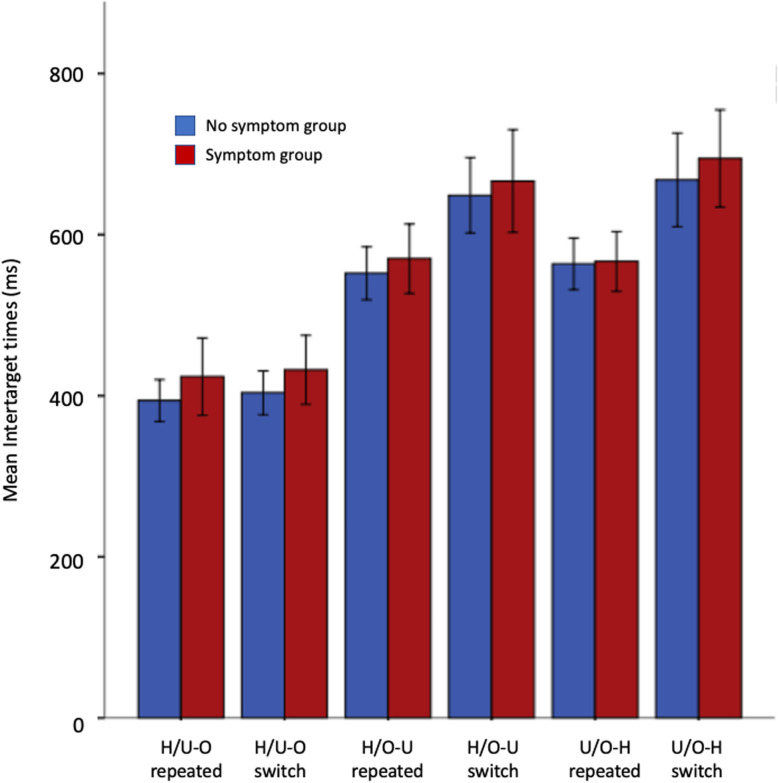
Mean ITT’s when observers selected targets from the same group or switched between groups, by condition and symptom groups

To further inspect switching within the two groups, a 3x2x2 (condition x switch x symptom group) repeated measure ANOVA was conducted on ITT’s when participants either switched between target categories or selected items from the same category. There was a significant main effect of condition (*F*(2,84) = 255.75, *p* < .001, η_p_^2^ = .859) and switching (*F*(1,42) = 98.46, *p* < .001, η_p_^2^ = .701) but the main effect of symptom group (*F*(1,42) = .662, *p* = .421, η_p_^2^ = .016) was not significant. There was also a significant interaction between condition and switch condition (*F*(2,84) = 36.6, *p* < .001, η_p_^2^ = .466). No other interactions were significant (all *p’s* > .575). Figure 5 shows that the mean ITT’s were shortest in the H/U-O condition in both groups, both when observers repeat a target within the same category and when they switched between categories, consistent with what we have seen in previous analyses.

#### Regression analyses

We conducted a stepwise multiple regression to investigate whether scores on the self-report questionnaires predicted participants’ performance on the iPad foraging task. The best model for each dependent variable was chosen with the adjusted R^2^ value with collinearity diagnostics taken into account. The independent variables were the self-report scales. All significant models, the predictors in each model, the standardized coefficients and significance tests can be seen in Table 3.

**Table 3:** Significant models and coefficients from the regression analyses

Dependent variable		*B*	β	*T*	*P*
ITT (H/O-U)	Constant	770.1		12.5	.00
	BSQ	−3.51	−1.10	−2.74	.01
	EDE-Q shape concern	110.44	1.62	2.74	.01
	EDE-Q eating concern	78.8	.752	2.62	.013
	EDE-Q total	− 112	−1.25	−2.42	.021
NOR (H/U-O)	Constant	10.42		79.36	.00
	BES	.031	.418	2.8	.008
SW-C (H/U-O)	Constant	−2.38		−.275	.785
	EDE-Q restraint	14.4	.445	2.4	.02
	EDE-Q eating concern	−12.4	−.303	−1.67	.104

Note. *B* Unstandardized beta; *β* Standardized beta; *NOR* Number of runs; *SW-C* Switch-cost

The best model did not explain a significant part of the variance in ITT’s in the H/U-O and the O/U-H conditions (both adjusted R^2^ < .03, both *p’s* > .287). In the H/O-U condition the best model was significant (F(4,34) = 2.69, *p* = .047) and the model explained 15.1% of the variance in ITT’s and had a condition index of 24.1 in the collinearity diagnostics (see Table 3). The best model did not explain significant parts of the variance in run number in the H/O-U and the O/U-H conditions (both adjusted R^2^ < .081, both *p* > .119). In the H/U-O condition the best model was significant (*F*(1,37) = 7.83, *p* = .008), explaining 15.2% of the variance in run number and had a condition index of 3.47 in the collinearity diagnostics (Table 3). Finally, the best model did not explain a significant part of the variance in switch-costs in the H/O-U and the U/O-H conditions (both adjusted R^2^ < .015, both *p* > .288). In the H/U-O condition the best model was marginally significant (*F*(2,36) = 3.08, *p* = .058), explaining 10% of the variance in switch-cost with a condition index of 3.44 in the collinearity diagnostics (Table 3).

The regression analyses showed that our predictors explained some of the variation in intertarget times in the H/O-U condition, run numbers in the H/U-O condition and the prediction was marginally significant for switch costs in the H/U-O condition. While the regression results are not decisive enough for drawing strong conclusions, they are encouraging in that they suggest that performance on the foraging task may vary by measures of eating disorders.

#### Extreme group analysis

It is possible that our sample did not include many participants falling at the extremes of the diagnostic scales that we used. For example, one estimate puts the prevalence of eating disorders at 7 to 8% [22], so we may expect that only 3–4 of our 44 participants will actually have an eating disorder. Our 2-group split that included everyone in the sample may therefore not have been quite sensitive enough to differences between participants. In such cases, extreme group analyses (EGA) may be of use and are often used in research on psychopathology (see e.g. [21, 43]). In an exploratory analysis we selected the 8 participants that had the highest and lowest scores (combined) on our 4 measures of eating disorder symptoms and measured the difference between them on intertarget times, run numbers and switch costs (Table 4). There are no strict guidelines available for assignment to extreme groups but our selection of 8 participants for each group was based on scatterplot analysis of the results.

**Table 4:** Results of extreme group analyses, showing differences on each foraging measure between the extreme groups on each eating disorder scale

	Intertarget times				Run number				Switch costs			
	HO – U	HU-O	UO-H	Total	HO - U	HU-O	UO-H	Total	HO – U	HU-O	UO-H	Total
BSQ	23	34	39	31	− 0.4	0.3	− 0.6	− 0.4*	1	33	31	20
BES	57*	92**	79*	76**	0.5	0.4	0.2	0.1	−1	66**	21	29*
BUL	50	68*	95*	71**	0.2	0.4	0.4	0.2	6	105	140	84
EDEQ	45	49	57	51	0	−0.4	0.3	0.2	5	35	35	25

* *p* < .05

** *p* < 0.1

In the original analysis we found that the symptom group had longer mean ITT’s for all switches from food item to either another food item or object (H to U, H to O, U to H, U to O) but this pattern was never statistically significant. With the extreme group analysis, however, we find that the differences for the groups are in many cases significant for the BES and BUL scales. This indicates that foraging for food related stimuli may vary by participants outcomes on standardized indicators of eating disorders. We emphasize strongly that these extreme group analyses are post-hoc and highly exploratory and can only be used as hints for future research directions rather than strong evidence in favor of particular hypotheses. Nevertheless we do believe that they do provide encouragement for further study of foraging for food related items in relation to eating disorders, especially when we are dealing with at-risk groups.

## Discussion

Our aim was to assess the relationship between attentional bias to food items and eating disorder symptoms measured with self-report scales, using a modified version of the iPad foraging task created by Kristjánsson et al. [30]. To our knowledge this method has not been used previously to measure attention bias in eating disorders, nor for other clinical anxiety disorders. We hypothesized that participants experiencing eating disorder symptoms would show different foraging behavior than participants without any eating disorder symptoms. Previous findings have not yielded any consensus on the effects that eating disorder symptoms have on attentional bias to food and allocation of attention to food or other stimuli like body-weight or body-shape stimuli. We therefore did not quite know what result patterns to expect. It seems possible that the eating disorder symptom group could either allocate their attention *toward* food stimuli to a larger degree than the no symptom group, showing lower inter-target times than the no symptom group, or the symptom group might attend *away* from food stimuli, yielding higher-inter target times. Even though the differences between the two groups were not statistically significant in most of our analyses, there seemed to be an emerging consistent pattern of differences between the groups. Many of these patterns then became significant when we focused on the most extreme cases in the extreme group analysis. We note, however, that such post-hoc extreme group analyses must most certainly be taken with a grain of salt (see e.g. [21, 43]) and should be used as hints for future research directions rather than evidence in favor of particular hypotheses.

We focused on three measures, based on our previous foraging findings: intertarget times, run behavior and switch costs. Firstly the symptom group had higher *inter-target times* in all three conditions. For *switch costs*, the symptom group had longer mean ITT’s for all switch-types that involved switches from food item to either another food item or object (H to U, H to O, U to H, U to O). When switching from object to a food item (O-H, O-U), the groups either did not differ or the no symptom group had longer mean ITT’s. This suggests that participants with eating disorder symptoms may become “stuck” on the food items and may have difficulties disengaging attention away from the food items. These results are consistent with theories that suggest that anxiety provoking and threatening stimuli in our surroundings capture our attention and tend to hold onto it (e.g. [5, 12]). Thirdly, we assessed run behavior which seemed not to differ much between the groups.

Most of the best models that the regression analysis generated did not yield statistically significant outcomes on performance, and those that were significant did not explain particularly large parts of the variance in foraging performance. The EDE-Q and some of its subscales seemed to be the best predictor of foraging patterns of all the self-report questionnaires; the EDE-Q was included in the best model both when predicting ITT in the H/O-U condition and when predicting switch-costs in the H/U-O condition.

Notably, the H/U-O condition distinguished itself clearly from the other two conditions with the fastest ITT’s and the lowest switch costs. This suggests that there is a clear category effect for food items, since in this condition, observers had to distinguish food items from non-food items in general. These results are definitely encouraging for further development of food-based foraging tasks for research on eating disorders, although sensitivity should clearly be improved in terms of detecting potential attentional biases. Both the symptom and the no symptom group had the lowest mean ITT’s, larger run numbers and barely any switch costs in the H/U-O condition. This suggests that participants switch between food items more efficiently and effortlessly than between food items and other items. Many participants may make this categorization and view the two target categories in the H/U-O condition as one food target category which can explain the high number of runs and low switch costs in this condition. Meanwhile, the difference between the two target categories in the other conditions (healthy food items and objects, unhealthy food items and objects) is more clear-cut and participants may treat the two categories as separate. The run numbers in both groups in all conditions were similar to what has previously been seen for easy feature foraging, where only one feature distinguishes between the targets (Á [30, 37, 38].). Those results are perhaps surprising, as there are many different features that distinguish the targets, but importantly this shows how category-based selection can be utilized for efficient foraging.

### Future work

For a clearer picture of the effects eating disorder symptoms may have on foraging behavior a similar study should be performed in a similar sample but with more participants, yielding more statistical power for our analyses. There was a clear variance in participants score with about half the participants scoring above the designated cut off score on any of the questionnaires, but this was not represented equally on all scales with 14 participants scoring above the cut-off score on the restraint subscale on the EDE-Q but only 1 participant above the cut-off on the BULIT-R. Second, data should be gathered on participants with clear clinical diagnoses of eating disorders, and their performance compared with a non-clinical group. Thirdly, satiation levels are clearly an important variable that should be addressed in future research, one that we had no control over in our current study.

Our results are in many respects promising with regard to how feasible it is to assess potential biases to food related stimuli with tasks involving foraging for multiple targets. We note, for example that the category effects that we see between food items and other items are highly interesting. There was a strong effect of food versus non-food items on error rates, intertarget times and run numbers. This is encouraging in the long run for our project since a category effect of food items is important for any potential use of the foraging task for attention bias modification. Other paradigms for assessing attention bias typically involve single targets during visual search, or trials involving a single attention deployment following a cue (see [49–51] for review). The foraging paradigm is very efficient since it enables the gathering a lot of information in a short amount of time. The paradigm yields many informative dependent variables (see [34] for a recent review) and can therefore provide a multifaceted picture of potential attention biases.

Even though our results were not conclusive in terms of statistical significance, except when extreme group analyses were used, there was a relatively consistent pattern of differences that emerged between the symptom group and the no symptom group. We therefore expect that with a clinical sample and more variance in scores on the self-report questionnaires, the paradigm can provide insights into whether attentional biases are involved in a particular food-related pathology. The long-term goal of research on visual attention is to understand visual attention in a general sense but also in a more idiosyncratic way, and part of this involves understanding how particular biases affect attentional orienting. One of the most basic functions of vision and visual attention is to aid in the gathering of food, and attentional biases towards or away from particular types of food can in some cases become extremely dysfunctional. Investigating such food-related biases may in the end serve to improve diagnoses and also improve treatment through methods such as attentional bias modification.

## Conclusions

Our results suggest that our newly designed foraging task is sensitive to attentional biases related to eating disorders and as such has the potential for enabling assessment of eating disorders. It is important to note, however, that some of the evidence for this comes from post-hoc extreme group analyses. In addition, we speculate that the task may prove to be useful for the treatment of such disorders through attentional bias modification. It is, however, also important to note that further development in assessing differences between the groups is absolutely necessary before any such applications are made.

## Data Availability

All data and materials are available upon request from the corresponding author.

## Abbreviations

AN: Anorexia Nervosa
BN: Bulimia Nervosa

## References

[1] American Psychiatric Association (2013). Diagnostic and statistical manual of mental disorders.

[2] Arcelus J, Mitchell AJ, Wales J, Nielsen S (2011). Mortality rates in patients with anorexia nervosa and other eating disorders. Arch Gen Psychiatry.

[3] Baldofski S, Lüthold P, Sperling I, Hilbert A (2018). Visual attention to pictorial food stimuli in individuals with night eating syndrome: an eye-tracking study. Behav Ther.

[4] Beard C, Sawyer AT, Hofmann SG (2012). Efficacy of attentional bias modification using threat and appetitive stimuli: a meta-analytic review. Behav Ther.

[5] Beck AT (1976). Cognitive therapy and the emotional disorders.

[6] Bar-Haim Y, Lamy D, Pergamin L, Bakermans-Kranenburg MJ, Ijzendoorn MH (2007). Threat-related Attentional Bias in anxious and nonanxious individuals : a meta-analytic study. Psychol Bull.

[7] Berman ES (2006). The relationship between eating self-efficacy and eating disorder symptoms in a non-clinical sample. Eat Behav.

[8] Bond AB (1982). The bead game: response strategies in free assortment. Hum Factors.

[9] Bond AB (2007). The evolution of color polymorphism: Crypticity, searching images, and apostatic selection. Annu Rev Ecol Evol Syst.

[10] Brand J, Masterson TD, Emond JA, Lansigan R, Gilbert-Diamond D (2020). Measuring attentional bias to food cues in young children using a visual search task: an eye-tracking study. Appetite.

[11] Brascamp JW, Blake R, Kristjánsson Á (2011). Deciding where to attend: priming of pop-out drives target selection. J Exp Psychol Hum Percept Perform.

[12] Clark DM (1999). Anxiety disorders: why they persist and how to treat them. Behav Res Ther.

[13] Cooper PJ, Taylor MJ, Cooper Z, Fairburn CG (1987). The development and validation of the body shape questionnaire. Int J Eat Disord.

[14] Cooper Z, Fairburn CG (1987). The eating disorder examination: a semi-structured interview for the assessment of the specific psychopathology of eating disorders. Int J Eat Disord.

[15] Dawkins M (1971). Shifts of ‘attention’ in chicks during feeding. Anim Behav.

[16] Delago MM, Jacobs LF (2017). Caching for where and what: evidence for a mnemonic strategy in a scatter-hoarder. R Soc Open Sci.

[17] Desimone R, Duncan J. Neural mechanisms of selective visual attention. Annu Rev Neurosci. 1995;18(1):193–222. 10.1146/annurev.ne.18.030195.001205 7605061

[18] Dobson KS, Dozois DJA (2004). Attentional biases in eating disorder: a meta-analytic review of Stroop performance. Clin Psychol Rev.

[19] Eisenberg D, Nicklett EJ, Roeder K, Kirz NE (2011). Eating disorder symptoms among college students: Prevelance, persistence, correlates and treatment-seeking. J Am Collage Health.

[20] Faunce GJ (2002). Eating disorders and attentional bias: a review. Eat Disord.

[21] Fisher JE, Guha A, Heller W, Miller GA (2020). Extreme-groups designs in studies of dimensional phenomena: advantages, caveats, and recommendations. J Abnorm Psychol.

[22] Galmiche M, Déchelotte P, Lambert G, Tavolacci MP (2019). Prevalence of eating disorders over the 2000–2018 period: a systematic literature review. Am J Clin Nutr..

[23] Gee A, Troop NA (2003). Shame, depressive symptoms and eating, weight and shape concerns in a non-clinical sample. Eat Weight Disord.

[24] Gormally J, Black S, Daston S, Rardin D (1982). The assessment of binge eating severity among obese persons. Addict Behav.

[25] Gunnarsdóttir OJ (2011). Lotugræðgi: hlutverk fullkomnunaráráttu og sjálfsálits (BS dissertation).

[26] Jónsdóttir SM, Þorsteinsdóttir G, Smári J. Próffræðilegir eiginleikar íslenskar gerðar Bulimia Test-Revised (BULIT-R) prófsins. Icelandic Med J. 2005;12:923–31. 16333153

[27] Kaye WH, Bulik CM, Thornton L, Barbarich N, Masters K (2004). Comorbidity of anxiety disorders with anorexia and bulimia nervosa. Am J Psychiatry.

[28] Konkle T, Brady TF, Alvarez GA, Oliva A (2010). Conceptual distinctiveness supports detailed visual long-term memory for real-world objects. J Exp Psychol Gen.

[29] Kristjánsson Á, Ásgeirsson ÁG (2019). Attentional priming: recent insights and current controversies. Curr Opin Psychol.

[30] Kristjánsson Á, Jóhannesson ÓI, Thornton IM (2014). Common attentional constraints in visual foraging. PLoS One.

[31] Kristjánsson T, Kristjánsson Á (2018). Foraging through multiple target categories reveals the flexibility of visual working memory. Acta Psychol.

[32] Kristjánsson Á, Mackeben M, Nakayama K (2001). Rapid, object-based learning in the deployment of transient attention. Perception.

[33] Kristjánsson Á, Nakayama K (2002). The attentional blink in space and time. Vis Res.

[34] Kristjánsson Á, Ólafsdóttir IM, Kristjánsson T. Visual foraging tasks provide new insights into the orienting of visual attention: methodological considerations. In: Neuromethods: Humana Press; 2019. 10.1007/7657_2019_21.

[35] Kristjánsson A. Rapid learning in attention shifts: A review. Visual Cognition. 2006:13(3):324–362.

[36] Kristjánsson Á, Sigurjónsdóttir Ó, Driver J. Fortune and reversals of fortune in visual search: Reward contingencies for pop-out targets affect search efficiency and target repetition effects. Attention, Perception, & Psychophysics. 2010;72(5):1229–36. 10.3758/APP.72.5.1229 20601703

[37] Kristjánsson T, Draschkow D, Pálsson Á, Haraldsson D, Jónsson PÖ, Kristjánsson Á. Moving foraging into three dimensions: feature- versus conjunction-based foraging in virtual reality. Q J Exp Psychol. 2020a. 10.1177/1747021820937020. 10.1177/1747021820937020 32519926

[38] Kristjánsson T, Thornton IM, Chetverikov A, Kristjánsson Á (2020). Dynamics of visual attention revealed in foraging tasks. Cognition.

[39] MacLeod C, Mathews A, Tata P (1986). Attentional bias in the emotional disorders. Research and Therapy.

[40] Mathews A, MacLeod C. Selective processing of threat cues in anxiety states. Behav Res Ther. 1985;23:563–9. https://doi.org/10.1016/0005–7967(85)90104–4. 10.1016/0005-7967(85)90104-4 4051929

[41] Nakayama K, Martini P. Situating visual search. Vision Res. 2011;51(13):1526–37. 10.1016/j.visres.2010.09.003 20837042

[42] Ólafsdóttir SM (2011). *Próffræðilegir eiginleikar íslenskrar þýðingar Eating Disorder Evaluation-Questtionnair (EDE-Q) og Clinical Impairment Assessment (CIA)* (Cand. Psych. Dissertation).

[43] Preacher KJ, Rucker DD, MacCallum RC, Nicewander WA (2005). Use of the extreme groups approach: a critical reexamination and new recommendations. Psychol Methods.

[44] Raymond JE, Shapiro KL, Arnell KM (1992). Temporary suppression of visual processing in an RSVP task: an attentional blink?. J Exp Psychol Hum Percept Perform.

[45] Rieger E, Schotte DE, Touyz SW, Beumont PJV, Griffiths R, Russell J (1998). Attentional biases in eating disorders: a visual probe detection procedure. Int J Eat Disord.

[46] Schmidt R, Lüthold P, Kittel R, Tetzlaff A, Hilbert A (2016). Visual attentional bias for food in adolescents with binge-eating disorder. J Psychiatr Res.

[47] Shafran R, Lee M, Cooper Z, Palmer RL, Fairburn CG (2007). Attentional Bias in eating disorders. Int J Eat Disord.

[48] Shurygina O, Kristjánsson Á, Tudge L, Chetverikov A (2019). Expectations and perceptual priming in a visual search task: evidence from eye movements and behavior. J Exp Psychol Hum Percept Perform.

[49] Sigurjónsdóttir Ó, Björnsson AS, Ludvigsdottir, & Kristjánsson, Á. (2015). Money talks in attention bias modification: reward in a dot-probe task affects attentional biases. Vis Cogn.

[50] Sigurjónsdóttir Ó, Sigurðardóttir S, Bjornsson AS, Kristjánsson Á (2015). Barking up the wrong tree in attentional bias modification? Comparing the sensitivity of four tasks to attentional biases. J Behav Ther Exp Psychiatry.

[51] Sigurjónsdóttir Ó, Bjornsson AS, Wessmann ID, Kristjánsson Á (2020). Measuring biases of visual attention: a comparison of four tasks. Behavioral Sciences.

[52] Smeets E, Roefs A, Furth EV, Jansen A (2008). Attentional bias for body and food in eating disorders: increased distraction, speeded detection, or both?. Behav Res Ther.

[53] Sperling I, Baldofski S, Lüthold P, Hilbert A (2017). Cognitive food processing in binge-eating disorder: an eye-tracking study. Nutrients.

[54] Stroop JR (1935). Studies of interference in serial verbal reactions. J Exp Psychol.

[55] Swinbourne JM, Touyz SW (2007). The co-morbidity of eating disorders and anxiety disorders: a review. Eur Eat Disord Rev.

[56] Tagu J, Kristjánsson Á. Dynamics of attentional and oculomotor orienting in visual foraging tasks. Q J Exp Psychol. 2020. 10.1177/1747021820919351. 10.1177/1747021820919351 32238034

[57] Talbot D, Smith E, Cass J (2019). Male body dissatisfaction, eating disorder symptoms, body composition, and attentional bias to body stimuli evaluated using visual search. Journal of Experimental Psychopathology.

[58] Thelen MH, Farmer J, Wonderlich S, Smith M (1991). A revision of the bulimia test: the BULIT—R. Psychological Assessment: A Journal of Consulting and Clinical Psychology.

[59] Thornton IM, de’Sperati, C. & Kristjánsson, Á. (2019). The influence of selection modality, display dynamics and error feedback on 55 patterns of human foraging. Vis Cogn.

[60] Thornton IM, Nguyen TT, Kristjánsson Á. Foraging tempo: human run patterns in multiple-target search are constrained by the rate of successive responses. Q J Exp Psychol. 2020. 10.1177/1747021820961640. 10.1177/1747021820961640 32933424

[61] Tinbergen L. The natural control of insects in pinewoods I. Factors influencing the intensity of predation by songbirds *Archives Néerlandaises de Zoologie*. 1960;13:265–34.

[62] Vigfúsdóttir J (2013). *Þýðing og próffræðilegir eiginleikar íslenskrar þýðingar Lotuofátslistans – Binge Eating Scale* (Cand. Psych. Dissertation).

[63] Williams JMG, Watts FN, MacLeod C, Mathews A (1997). Cognitive psychology and the emotional disorders.

[64] Williamson DA, White MA, York-Crowe E, Stewart TM (2004). Cognitive-behavioral theories of eating disorders. Behav Modif.

[65] Wolfe, J. M. (2013). When is it time to move to the next raspberry bush? Foraging rules in human visual search. J Vision*,* 13(3):10, 1-17. 10.1167/13.3.10 PMC4521330 23641077

[66] Wolfe JM, Cain MS, Alaoi-Soce A (2018). Hybrid value foraging: how the value of targets shapes behavior. Attention, Perception, and Psychophysics.

[67] Wolfe JM, Horowitz TS (2004). What attributes guide the deployment of visual attention and how do they do it?. Nat Rev Neurosci.

[68] Woods AJ, Göksun T, Chatterjee A, Zelonis S, Mehta A, Smith SE (2013). The development of organized visual search. Acta Psychol.

[69] Öhman A, Flykt A, Esteves F (2001). Emotion drives attention: detecting the snake in the grass. J Exp Psychol Gen.

[70] Ævarsdóttir D, Guðnadóttir M. *Próffræðilegir eiginleikar og tengsl við lystarstol og lotugræðgi* (BS dissertation). Reykjavík: University of Iceland; 2008.

